# Whole body computed tomography scanning for severe blunt polytrauma: analysis of Trauma Audit and Research Network database 2005 to 2010

**DOI:** 10.1186/cc11064

**Published:** 2012-03-20

**Authors:** PA Hunt, F Lecky, O Bouamra

**Affiliations:** 1 James Cook University Hospital, Middlesbrough, UK; 2 Hope Hospital, Salford, UK

## Introduction

There is growing evidence to recommend the use of whole body computed tomography (WBCT) scanning in the early management of severe blunt polytrauma patients. One recent study reported a survival advantage when using WBCT compared to a conventional imaging approach [1]. A number of UK NHS institutions already utilise WBCT protocols based upon either injury mechanism-related or physiological factors, or a combination of these. However, the UK Royal College of Radiologists is yet to provide recommendations on the use of WBCT in polytrauma. We present the results of our analysis of a large retrospective case series from 2005 to 2010 taken from the Trauma Audit and Research Network (TARN) database. We believe this is the first analysis of its kind involving UK trauma cases and provides important evidence to support the use of WBCT and guide best clinical practice.

## Methods

We utilised retrospective, multicentre data of severe blunt polytrauma (ISS >15) direct ED admissions aged >15 years recorded in the UK TARN database to compare survival at 30 days between two groups of patients: those who underwent WBCT scans, and those who received a focused CT scan approach as part of their initial management in the emergency department. A total of 12,792 cases were included in the final dataset.

## Results

A total 2,822 (22%) of 12,792 cases underwent WBCT from the ED. The median ISS for the WBCT group was 22 (IQR 14 to 33) compared to 16 (IQR 9 to 25) for the focused CT group. The calculated crude mortality rate for the WBCT group was 10.1% compared to 8.7% in the focused CT group (*P *= 0.0124). Multivariate analysis with adjustments for potential confounding factors demonstrated an OR of 1.313 (95% CI = 1.083 to 1.592, *P *= 0.006) in favour of survival in the WBCT group.

## Conclusion

Despite the crude mortality rates appearing to demonstrate a poorer outcome in the WBCT group, correcting for confounding factors revealed an around 30% improvement in survival for the WBCT group. However, when also correcting for the potential effect of clustering, the benefit of WBCT is less clear, with an around 20% improvement in survival and a lower level of significance (*P *= 0.084). This effect may, in part, be due to differing trauma systems and logistical organisation between institutions. Overall, the results of our investigation appear to suggest a potential survival benefit from the use of WBCT in severe blunt polytrauma.
